# Supplementary material for: A Global Screen for Assembly State Changes of the Mitotic Proteome by SEC-SWATH-MS
Source: Cell Syst. 2020 Feb 26;10(2):133–155.e6. doi: 10.1016/j.cels.2020.01.001 (PMC7042714; doi:10.1016/j.cels.2020.01.001)

P37802 | TAGL2_HUMAN | TAGLN2 KIAA0120 CDABP0035
Monomer MW [kDa]: 22.391 Monomer expected elution fraction: 53

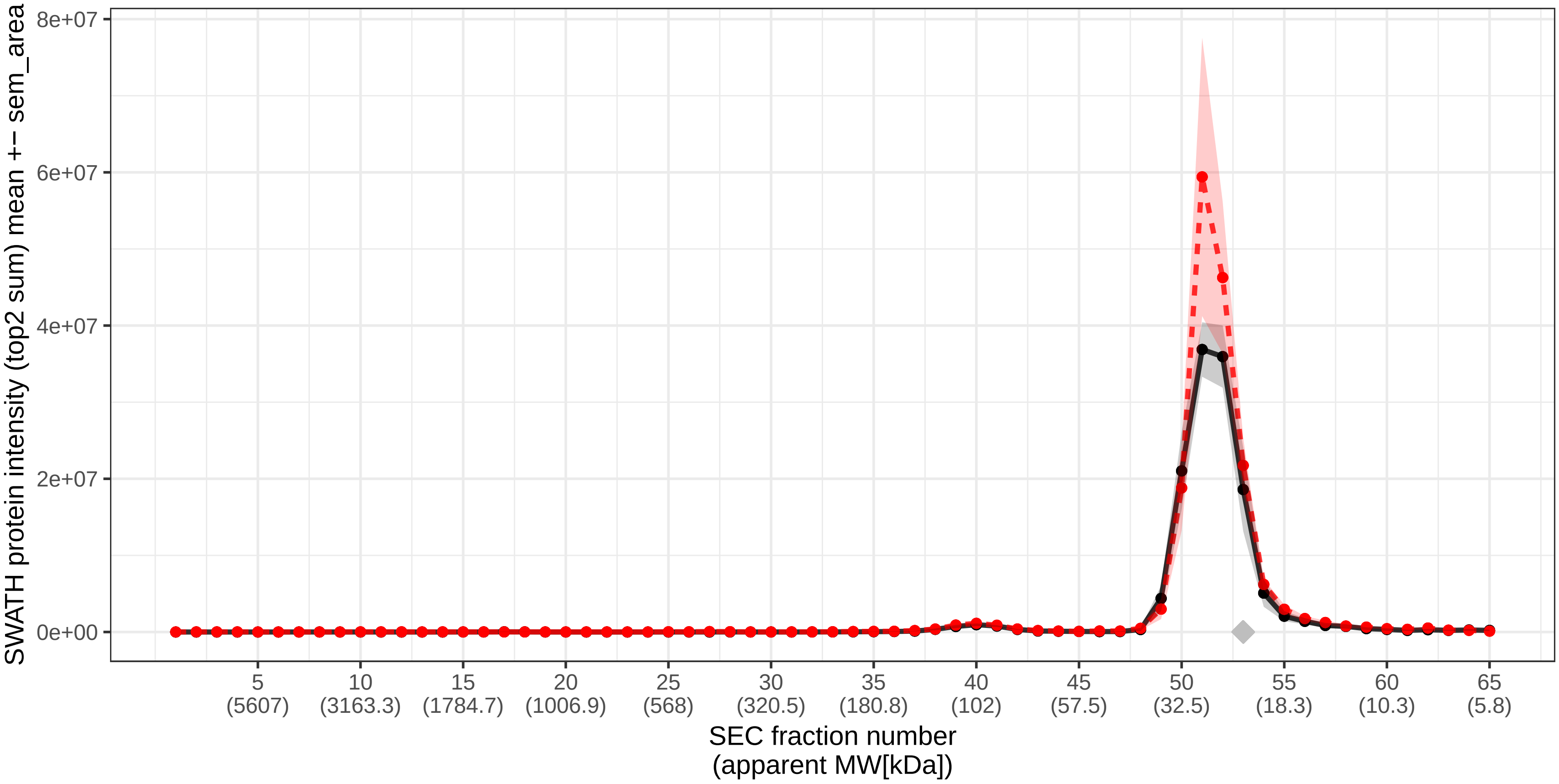

Supplement: Data S1. SEC-SWATH-MS Protein Chromatograms, Related to Figure 1 [file mmc6.zip › SECchrom_P37802_TAGL2_HUMAN_TAGLN2_KIAA0120_CDABP0035.pdf]
